# In Vitro Analysis of Selected Antioxidant and Biological Properties of the Extract from Large-Fruited Cranberry Fruits

**DOI:** 10.3390/molecules28237895

**Published:** 2023-12-01

**Authors:** Maciej Balawejder, Tomasz Piechowiak, Ireneusz Kapusta, Aleksandra Chęciek, Natalia Matłok

**Affiliations:** 1 Department of Chemistry and Food Toxicology, University of Rzeszow, St. Ćwiklińskiej 1a, 35-601 Rzeszow, Poland; mbalawejder@ur.edu.pl (M.B.); tpiechowiak@ur.edu.pl (T.P.); olach2000@wp.pl (A.C.); 2 Department of Food Technology and Human Nutrition, University of Rzeszow, St. Zelwerowicza 4, 35-601 Rzeszow, Poland; ikapusta@ur.edu.pl; 3 Department of Food and Agriculture Production Engineering, University of Rzeszow, St. Zelwerowicza 4, 35-601 Rzeszow, Poland

**Keywords:** *Vaccinium macrocarpon*, extract, bioactive compounds, antioxidants, SOD and CAT activity, anti-inflammatory properties, cyclooxygenase-1 (COX-1), cyclooxygenase-2 (COX-2), acetylcholinesterase (AChE)

## Abstract

This study investigated the in vitro antioxidant and biological properties of ethanol extracts obtained from the fruits of the highbush cranberry. The produced extracts exhibited a high content of polyphenols (1041.9 mg 100 g d.m.^−1^) and a high antioxidant activity (2271.2 mg TE g 100 d.m.^−1^ using the DPPH method, 1781.5 mg TE g 100 d.m.^−1^ using the ABTS method), as well as a substantial amount of vitamin C (418.2 mg 100 g d.m.^−1^). These extracts also demonstrated significant in vitro biological activity. Studies conducted on the *Saccharomyces cerevisiae* cellular model revealed the strong antioxidant effects of the extract, attributed to a significant reduction in the levels of reactive oxygen species (ROS) within the cells, confirming the utility of the extracts in mitigating oxidative stress. Moreover, inhibitory properties were demonstrated against factors activating metabolic processes characteristic of inflammatory conditions. It was observed that the cranberry extract inhibits the activity of cyclooxygenase-1 (COX-1) and cyclooxygenase-2 (COX-2) non-selectively. Additionally, the extract was found to be a highly active inhibitor of acetylcholinesterase (AChE), potentially suggesting the applicability of this extract in the prevention of neurodegenerative diseases, including Alzheimer’s disease.

## 1. Introduction

Berry fruits, including various species of *Vaccinium*, represent a rich source of bioactive compounds [1]. Both bog cranberries (*Vaccinium oxycoccos*), which are naturally occurring in the wild, and large-fruited cranberries (*Vaccinium macrocarpon*), which are extensively cultivated in the United States, Lithuania, Estonia, and Poland, boast a significant accumulation of bioactive compounds. The largest plantation of the latter species in Europe is situated in Poland [2,3,4,5].

*Vaccinium macrocarpon*, an evergreen shrub with creeping stems, is cultivated in peat bogs or sandy soils. It has high water requirements. Large-fruited cranberry fruits in Europe are typically mature in September and characterized by their red to dark red color, intensely acidic taste, and, depending on the variety, a round, oval, flattened, or cylindrical shape [3,6,7]. Fruits of this species are a rich source of phenolic compounds, primarily anthocyanins, flavonols, phenolic acids, and proanthocyanidins, to which are attributed antioxidant [8], anticancer, antimicrobial, antiangiogenic, and anti-inflammatory properties [9,10,11,12]. In vitro and in vivo studies indicate that extracts from large-fruited cranberry may exhibit anti-inflammatory effects through various mechanisms. Compounds like proanthocyanidins possess the ability to inhibit the activation of pro-inflammatory factors such as cytokines and enzymes involved in inflammatory responses. Additionally, consuming large-fruited cranberry fruits and their extracts could influence the regulation of signaling pathways related to inflammatory processes, potentially reducing the severity of inflammation. These fruits also contain significant amounts of ascorbic acid, which supports nervous system function, cell protection against oxidative stress, and proper collagen production [12]. Furthermore, they have been found to contain resveratrol, which impacts the cardiovascular system, including platelet aggregation inhibition and inflammation reduction [13]. Aside from their abundant bioactive components, large-fruited cranberry fruits are also characterized by low carbohydrate content compared to other fruits [14], making them a valuable component of the human diet.

Despite promising research results showcasing the health-promoting properties attributed to the presence of various phytochemical compounds in large-fruited cranberry, further work is necessary to fully understand the mechanisms of action of these fruits and to confirm their effectiveness in the context of inflammation prevention and therapy in humans.

The aim of this study was to analyze selected antioxidant and biological properties, including the antioxidant properties of an extract obtained from large-fruited cranberry fruits of the *Vaccinium macrocarpon* “Pilgrim” variety cultivated in Poland. The inhibitory activity of the cranberry extract against cyclooxygenase-1 (COX-1) and cyclooxygenase-2 (COX-2), which are mediators of inflammation, and acetylcholinesterase (AChE), which is responsible for acetylcholine breakdown and potentially linked to neurological disorders, was analyzed. Additionally, the antioxidant properties of the extract were determined using *Saccharomyces cerevisiae* yeast cells exposed to the toxic effects of H_2_O_2_.

## 2. Results and Discussion

### 2.1. Characteristics of Bioactive Substances in the Extract from Large-Fruited Cranberry Fruits

Large-fruited cranberry fruits are characterized by a high content of bioactive compounds, primarily flavonoids, especially proanthocyanidins, anthocyanidins, and flavonols, along with phenolic acids and benzoates [15]. These compounds exhibit diverse biological properties, particularly antioxidant effects. When inflammatory processes occur in higher organisms, elevated levels of reactive oxygen species (ROS) are recorded, which can be mitigated through the consumption of extracts containing high concentrations of readily available small-molecule antioxidants [16]. Among these compounds are polyphenols, with a content of 1041.9 ± 2.29 mg 100 g d.m.^−1^ in the obtained large-fruited cranberry extract (Table 1). In contrast, the total polyphenol content in fresh fruits is around 750 mg 100 g d.m.^−1^ on average [16]. Due to their tart taste, large-fruited cranberry fruits are rarely consumed raw. Additionally, to obtain an individual dose of polyphenols, one would need to consume roughly twice the mass of cranberries compared to the dry mass of the extract. Extracts can therefore serve as an equivalent to fresh large-fruited cranberry fruits, which are infrequently consumed in that form due to their sensory characteristics. However, culinary processing, especially thermal treatment, significantly impacts the decline of these thermally labile constituents [17]. The majority of harvested cranberries are further processed to make fruit juice and other cranberry food products; 35% are processed into sauce products and 60% are processed into various fruit drinks. Only 85% of the total cranberry is used for processing into juice, while the remaining 15% is processed into pomace [5].

Another valuable component of large-fruited cranberry fruits with antioxidant properties is vitamin C, with an average content of around 100 mg per 100 g fresh weight [16]. The obtained extracts exhibited a significantly higher content of this compound, exceeding 418 mg per 100 g fresh weight (Table 1). The daily requirement for this vitamin is around 100 mg, on average. This dosage is already provided by the consumption of 25 mg of the extract (calculated on a dry mass basis). On the other hand, in the case of fresh large-fruited cranberry fruits, which contain approximately 90% water [18], to meet the daily requirement for vitamin C, one would need to consume about 1 kg of the fruit.

Vitamin C and polyphenols, along with other compounds, collectively shape the overall antioxidant potential of plant materials. Depending on the applied measurement method, this potential ranged from 1781.5 (ABTS method) to 2271.2 (DPPH method) trolox equivalents per 100 g fresh weight of the extract (Table 1).

### 2.2. Profile of Polyphenolic Compounds in Cranberry Extract

Large-fruited cranberry fruits are a globally esteemed source of polyphenolic compounds. The content and profile of these compounds vary significantly and depend on various factors, primarily including the variety, habitat, and meteorological conditions, as well as the cultivation techniques employed [19].

**Figure 1:**
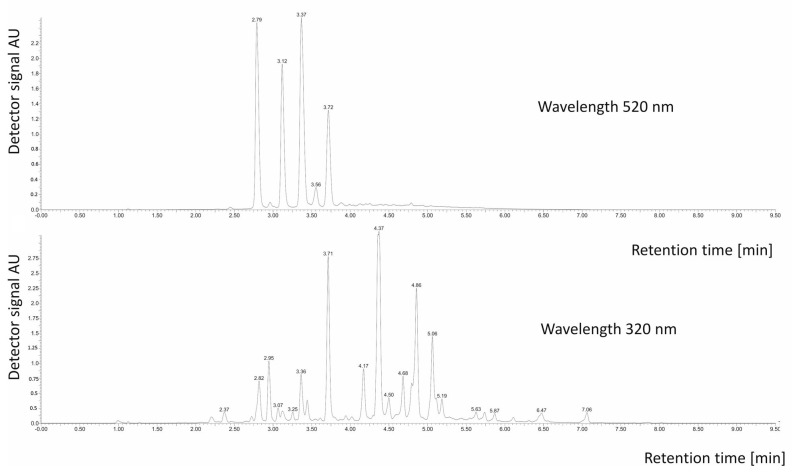
Chromatogram of phenolic compounds (350 nm) and anthocyanins recorded with a UV-VIS detector.

In the obtained extract from large-fruited cranberry fruits, 32 polyphenolic compounds were identified, including 8 anthocyanins responsible for fruit color (Table 2). The predominant anthocyanin in the examined extract was peonidin 3-O-glucoside, with a content of 97.1 mg per 100 g dry weight (Table 2 and Figure 1). In fresh large-fruited cranberry fruits, the contribution of this anthocyanin typically accounts for around 30% of the total anthocyanin content [20,21,22].

Among the other identified polyphenolic compounds in the large-fruited cranberry extract was quercetin 3-*O*-glucoside, with a content of 186.2 mg per 100 g fresh weight. The high content of this polyphenolic compound might contribute to the extract’s significant activity, as Prasain et al. [23] found that it had a potent inhibitory effect on the growth of human cancer cell lines. The majority of the identified polyphenolic compounds in the investigated extract exhibit diverse biological activities associated with antioxidant, anti-inflammatory, anti-cancer, anti-atherosclerotic, antimutagenic, antithrombotic, and antibacterial properties [24]. In many cases, there is a synergy among bioactive compounds, leading to enhanced activity that exceeds the sum of the individual components’ activities. Most of the remaining identified polyphenolic compounds are also present in fresh large-fruited cranberry fruits, albeit in significantly lower quantities [19].

### 2.3. Profile of Volatile Compounds in the Obtained Cranberry Extract

**Figure 2:**
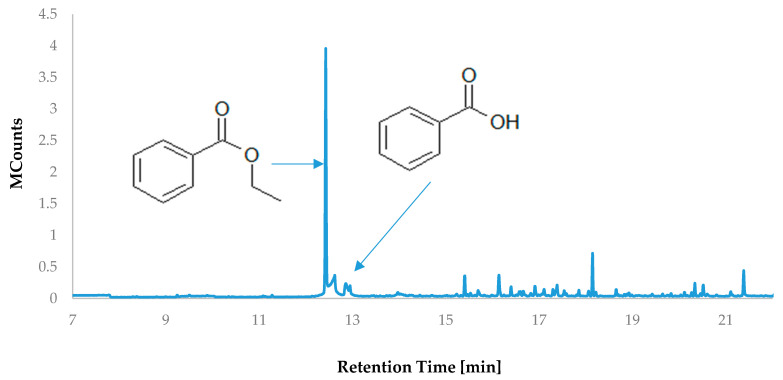
Chromatogram of volatile compounds isolated from large-fruited cranberry fruit extract.

Volatile organic compounds play a significant role in shaping the taste and aroma perceptions of food products [25]. Their presence arises from the raw material content or is a result of transformations linked to the applied processing technology. The majority of volatile substances are low-polarity small-molecule compounds, which notably differentiates them from polyphenolic compounds [26]. The most prevalent compound in the headspace volatile fraction of HS-VOC cranberry fruit extract was ethyl benzoate, formed through the esterification of ethanol (the extraction solvent) with benzoic acid (Table 3; Figure 2). This indicates a substantial content of this acid in the extract; however, HS-SPME analysis revealed trace amounts due to its significantly lower vapor pressure compared to the ethyl ester. The vapor pressure of organic compounds is closely dependent on substance polarity. In the case of carboxylic acids, their polarity is much higher than that of their corresponding esters [27]. Furthermore, carboxylic acids, including benzoic acid, form stable dimers in solutions, greatly reducing their volatility [28]. The remaining volatile compounds belong to the group of terpene hydrocarbons and their oxygenated derivatives. The highest contribution in the mixture, accounting for 14.66%, was β-caryophyllene, which was also identified in cranberry fruits [29]. De Lange et al. [29] identified the genes responsible for the biosynthesis of this compound group in cranberries, particularly the synthase of α-humulene, β-caryophyllene, and (3S,6E)-nerolidol/R-linalools.

### 2.4. Inhibitory Activity against COX-1, COX-2, and AChE

**Figure 3:**
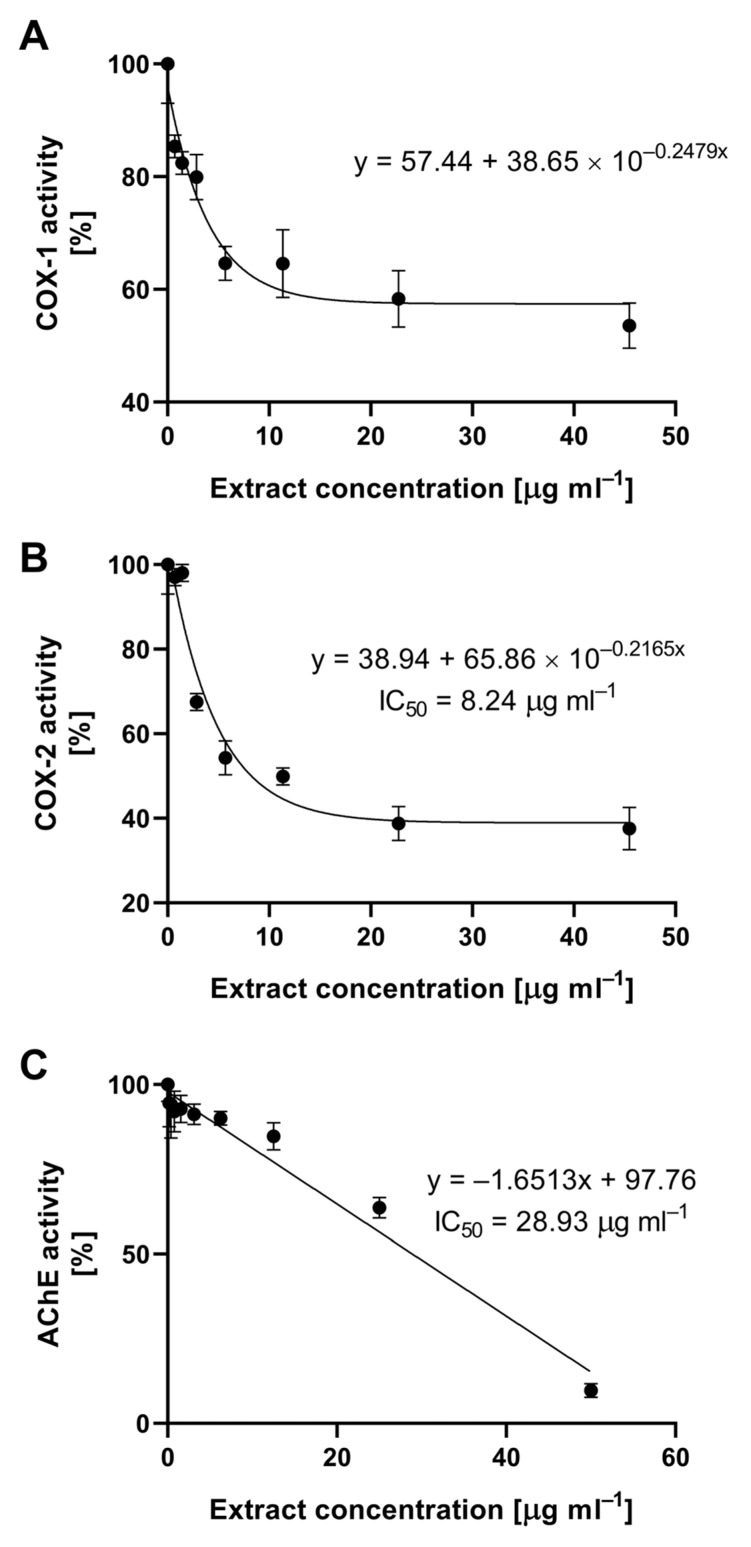
Inhibition of cyclooxygenase 1 (**A**), cyclooxygenase 2 (**B**), and acetylcholinesterase (**C**) by cranberry fruit extract.

Inflammatory conditions often accompany pathological changes in organisms, with enzymes cyclooxygenase-1 (COX-1) and cyclooxygenase-2 (COX-2) serving as their mediators. These enzymes catalyze the transformation of phospholipids in the cell membrane, leading to the production of prostanoids, including prostaglandins (PG), prostacyclins (PGI), and thromboxanes (TXA) [30]. The inhibition of the activity of these enzymes, especially COX-2, is a pharmacological mechanism employed to combat inflammation. Synthetic compounds exhibiting such activity belong to the group of non-steroidal anti-inflammatory drugs (NSAIDs), which include acetylsalicylic acid (aspirin), indomethacin, and ibuprofen [31]. Synthetic pharmaceuticals are designed to be highly selective in inhibiting the COX-2 enzyme, the activity of which is closely linked to the presence of inflammation. Extracts obtained from large-fruited cranberry fruits did not exhibit such selectivity, but demonstrated significant activity. The inhibitory activity of the tested extract against cyclooxygenase 1 and 2 is shown in Figure 3. The relationship between extract concentration and the activity of COX-1 and COX-2 followed an exponential trend. The concentration causing a 50% decrease in COX-2 activity was 8.24 µg mL^−1^. In the case of COX-1, it was not possible to calculate the IC50 value because a maximum inhibition level of 57.44% was achieved at the highest concentration.

Acetylcholinesterase (AChE) is an enzyme that breaks down one of the fundamental neurotransmitters, acetylcholine. This process is crucial for the functioning of the nervous system, and its disruption is a mechanism in many neurodegenerative diseases, including Alzheimer’s disease [32]. Since the late 1990s, intensive research has been conducted on specific, fully reversible acetylcholinesterase inhibitors for their potential use in mitigating the effects of this severe civilization-related disease. The cranberry extract obtained showed inhibitory action, and the relationship between its concentration and the decrease in acetylcholinesterase AChE activity was linear. The concentration causing a 50% decrease in AChE activity was already 28.93 µg mL^−1^ of cranberry fruit extract, as determined under in vitro conditions (Figure 3).

The results regarding the activities of COX-1, COX-2, and AChE highlight the complex activity of cranberry fruit extract, which could potentially offer an alternative to pharmaceutical products in combating inflammatory conditions and neurodegenerative diseases.

### 2.5. Evaluation of Antioxidant Properties of Extract Using S. cerevisiae Yeast Exposed to Toxic Effects of H_2_O_2_

**Figure 4:**
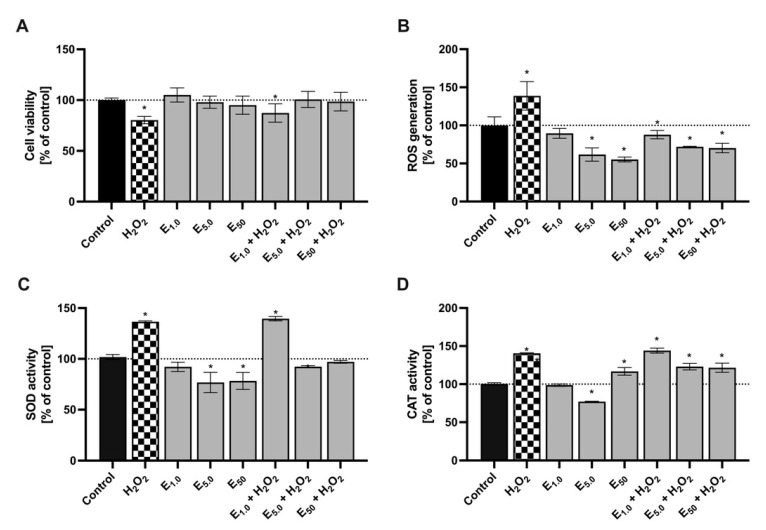
The ability of cranberry fruit extract to reduce oxidative stress in *S. cerevisiae* exposed to hydrogen peroxide. Average values denoted by “*” are statistically significantly different at α = 0.05 compared to the control. (**A**) yeast metabolic activity. (**B**) yeast cell capability for reactive oxygen species generation. (**C**) superoxide dismutase activity in yeast cells. (**D**) catalase activity in yeast cells.

*S. cerevisiae,* commonly known as baker’s yeast, is a frequently used model organism in research on the antioxidant properties of phytochemicals. This is due to the homology of certain defensive mechanisms against oxidative stress in higher organisms, including humans, as well as the ease of conducting cultivation [33]. In the present study, the impact of enriching *S. cerevisiae* culture with cranberry extract at concentrations of 1, 5, and 50 μg mL^−1^ was determined. Additionally, these cultures were exposed to an oxidative stress-inducing agent (hydrogen peroxide). The results of these experiments are depicted in Figure 4.

As expected, subjecting *S. cerevisiae* cells to hydrogen peroxide clearly induced oxidative stress. This was manifested by a slower growth rate, as measured by the Alamar Blue assay (20% reduction), as well as significantly higher levels of intracellular reactive oxygen species (ROS) levels (39% increase) and an elevated activity of antioxidant enzymes, such as superoxide dismutase (SOD) (37% increase) and catalase (CAT) (41% increase), compared to the control.

Throughout the investigation, it was shown that the introduction of cranberry extract counteracted the oxidative stress induced by hydrogen peroxide. However, this effect was observed only at concentrations of 5 and 50 μg mL^−1^. Yeast cells treated with both H_2_O_2_ and the extract displayed ROS levels comparable to the control, along with similar SOD and CAT activity. Furthermore, the reduction in oxidative stress by antioxidants present in the extract improved cell viability and metabolic activity. Interestingly, the cranberry extract decreased ROS production in cells not treated with H_2_O_2_. At the highest concentration (50 μg mL^−1^), the ROS level in yeast decreased by 44.8%, and at the concentration of 5 μg mL^−1^, it decreased by 38.3% compared to the control. This confirmed the cell’s ability to metabolize the extract and its potent antioxidant properties.

## 3. Materials and Methods

### 3.1. Research Material

The research material consisted of cranberry (*Vaccinium macrocarpon* Aiton, family: *Ericaceae*) fruit of the “Pilgrim” variety (~20 kg) sourced from cultivation in Poland on sandy soil. The fruits were harvested in the first decade of October, at the collective ripening stage. The collected cranberries exhibited the dark red color typical of these fruits and had a water content of approximately 89%.

### 3.2. The Procedure for Preparing and the Yield of Extracts

To perform the extraction process, 500 g of blindly sampled fruit and 2.5 L of 96% ethanol were placed in a 3 L glass beaker. The extraction process was carried out for 48 h, resulting in a solution with an intense red color. After this time, plant tissues were separated via filtration using filter paper. The obtained clear extract underwent a distillation process under reduced pressure (55 °C, 150 mbar), removing 90% of the solvent volume. As a final result, a viscous liquid with intense color and a characteristic cranberry aroma was obtained. A 50 mL of the extract was dried to determine the yield, which was 8%.

### 3.3. The Total Polyphenol Content, Antioxidant Activity, and Vitamin C Content

To assess the total polyphenol content, vitamin C content, and antioxidant properties, a 1% aqueous solution of the dry mass of the extract was prepared. After centrifugation at 12,000× *g* for 15 min, the extract was used for analysis.

The total polyphenol content was measured using the Folin–Ciocalteu reagent, and the results were expressed as gallic acid equivalents per 100 g of dry extract mass. Antioxidant activity was tested using synthetic DPPH and ABTS radicals, and the results were expressed as trolox equivalents per 100 g of extract [34]. Vitamin C content was measured using a photometric method with 2,6-dichlorophenolindophenol, as described by Piechowiak et al. [35]. The above analyses were performed in triplicate.

### 3.4. Profile of Phenolic Compounds in Cranberry Extracts

Determination of polyphenolic compounds in the produced ethanolic extract of large-fruited cranberries was carried out using ultra-performance liquid chromatography (UPLC) on a Waters ACQUITY system (Waters, Milford, MA, USA), following the methodology described in the study by Matłok et al. [36]. The analyses were performed in triplicate.

### 3.5. Volatile Compound Profile of Cranberry Extracts

The volatile compound profile of the produced cranberry extracts was determined using the Headspace Solid-Phase Microextraction (HS-SPME) method with a 100 µm polydimethylsiloxane (PDMS) fiber from Supelco Ltd. (Bellefonte, PA, USA), following the methodology described in the study [36]. Fiber exposure was carried out using the headspace approach for 30 min at a temperature of 20 °C. The composition of desorbed compounds was analyzed using a Varian 450 GC gas chromatograph with a Varian 240 MS mass spectrometer (Varian, Palo Alto, CA, USA). The analyses were conducted in triplicate.

### 3.6. Inhibition of Cyclooxygenase-1 (COX-1), Cyclooxygenase-2 (COX-2), and Acetylcholinesterase (ACHE) by Cranberry Extract

Inhibition of human COX-1 and COX-2 by cranberry extracts was assessed using commercial kits from Cayman (catalog numbers: 701070, 701080). The assays involved incubating COX-1 or COX-2 with cranberry extract over a concentration range of 0.7–45 µg mL^−1^, along with arachidonic acid, followed by measurement of the produced prostaglandin F2α through enzyme-linked immunosorbent assay (ELISA) by monitoring absorbance at 405 nm. The inhibitory activity of the extracts against acetylcholinesterase was determined following the protocol of a commercial kit from Sigma-Aldrich (Burlington, MA, USA) (catalog number: MAK324). The measurement was based on the kinetic evaluation of the amount of hydrolyzed acetylcholine iodide with DTNB, in the presence of extracts at concentrations ranging from 0.195 to 50 µg mL^−1^. Inhibition of COX-1, COX-2, and AChE enzymes was expressed as a percentage of activity compared to the control (mixture without extract).

### 3.7. Antioxidant Properties Using Saccharomyces Cerevisiae

#### 3.7.1. Metabolic Activity

Prior to conducting the assessment of antioxidant properties, the cranberry extract at a concentration of 10 mg mL^−1^ was sterilized using a syringe filter with a pore size of 0.22 µm. The evaluation of antioxidant properties was performed using the NCPF 3178 yeast strain. Initially, yeast cells were cultured for 10 h at a temperature of 28 °C with shaking (150 rpm) in a medium containing 1% peptone, 2% glucose, and 1% yeast extract (YPD). The metabolic activity of yeast cells in cultures containing H_2_O_2_ (or 0.9% NaCl in the control) and cranberry extracts (or 0.9% NaCl in the control) was assessed using the Alamar Blue assay according to the protocol presented by Piechowiak et al. [35]. The final concentration of H_2_O_2_ was 0.0125 mM, while the concentrations of cranberry extracts were 1, 5, and 50 µg mL^−1^. The results of metabolic activity were expressed as a percentage of the control.

#### 3.7.2. Intracellular ROS Levels

A total of 1.8 mL of yeast suspension with OD_600_ = 0.1 was pipetted into a 12-well plate. Subsequently, H_2_O_2_ and the extract were added to achieve the above-mentioned concentrations. The plate was incubated for 6 h at 28 °C, after which the suspension was centrifuged at 5000× *g* for 5 min. The pellet was washed twice with PBS buffer and then resuspended in PBS containing 0.1% glucose and 5 µM 2′,7′-dichlorodihydrofluorescein diacetate to achieve an OD_600_ = 0.5. The suspension was transferred six times (100 µL each) to a black 96-well plate, followed by measuring the fluorescence kinetics at an excitation of 495 nm and emission of 520 nm at 28 °C [35].

#### 3.7.3. The Activity of Antioxidant Enzymes

The obtained pellet was suspended in 990 µL of PBS containing 0.5 M mannitol, and then 10 µL of lyticase (2 mg mL^−1^) was added. Cell wall digestion was carried out at 37 °C for 30 min, followed by centrifugation of the resulting spheroplasts at 5000× *g* for 5 min. The pellet was then resuspended in 500 µL of PBS containing a mixture of protease inhibitors, followed by sonication for 10 s on ice. Superoxide dismutase (SOD) activity in the lysates was measured using the adrenaline method. One unit of SOD activity was defined as the amount of protein that causes a 50% inhibition of adrenaline oxidation. Catalase activity was determined using a photometric method based on the colorimetric measurement of residual hydrogen peroxide with ammonium metavanadate [34]. Enzymatic activity was standardized to 1 mg of protein, which was quantified using the Bradford method.

### 3.8. Statistical Analysis

One-way analysis of variance (ANOVA) was conducted, at a significance level α = 0.05, using STATISTICA 13.1 software (TIBCO Software Inc., Hillview Avenue, Palo Alto, CA, USA). The mean values calculated from the three independent replications were analyzed statistically by comparing the results between the variants of the experiment.

## 4. Conclusions

American cranberry fruits are a rich source of bioactive compounds. These compounds can be efficiently extracted and concentrated, resulting in an extract characterized by high polyphenol content (1041.9 mg per 100 g dry weight), strong antioxidant potential (2271.2 mg TE per 100 g dry weight by DPPH method, 1781.5 mg TE per 100 g dry weight by ABTS method), and a significant amount of vitamin C (418.2 mg per 100 g dry weight). These extracts exhibit substantial biological activity.

In vitro studies have demonstrated the inhibitory properties of these extracts on factors involved in metabolic processes associated with inflammation. The cranberry extract has been found to non-selectively inhibit the activity of COX-1 and COX-2 enzymes. Additionally, the extract has demonstrated the potent inhibition of AChE, suggesting its potential utility in the prevention of neurodegenerative diseases, including Alzheimer’s disease.

Furthermore, the cytoprotective effects of the extracts were investigated in relation to the reduction in oxidative stress, particularly induced by H_2_O_2_. The yeast model organism, *S. cerevisiae,* was employed to assess the level of reactive oxygen species (ROS) and the enzymatic activity of the stress markers CAT and SOD. The addition of American cranberry fruit extract significantly lowered ROS levels in H_2_O_2_-exposed *S. cerevisiae* cells, leading to the modulation of SOD and CAT activity. The demonstrated in vitro biological activity of the obtained American cranberry fruit extracts suggests their potential usefulness in the prevention of diseases associated with oxidative stress and elevated cyclooxygenase and acetylcholinesterase activities.

The demonstrated significant activity towards the inhibition of COX and AChE under the proposed conditions suggests that future research on cellular or animal models would be useful to confirm the effectiveness of cranberry extracts in inhibiting some diseases, especially neurodegenerative diseases.

## Figures and Tables

**Table 1:** Characteristics of bioactive compounds in extract from large-fruited cranberry fruits.

Ascorbic acid (mg 100 g d.m.^−1^)	418.2 ± 21.4
Total phenolic compounds (mg 100 g d.m.^−1^)	1041.9 ± 2.29
Antioxidant activity against DPPH radicals (mg TE 100 g d.m.^−1^)	2271.2 ± 7.31
Antioxidant activity against ABTS radicals (mg TE 100 g d.m.^−1^)	1781.5 ± 24.9

**Table 2:** Individual phenolic compounds identified using UPLC-PDA-MS/MS.

Compound		Rt	λ_max_	[M-H]^+/−^ *m*/*z*		Content (mg 100 g d.m^.−1^)
		min	nm	MS	MS/MS	
*Anthocyanins*						
1	Peonidin *O*-hexoside	2.44	279,524	463^+^	301	1.15 ± 0.07
2	Cyanidin 3-*O*-glucoside	2.80	279,512	449^+^	287	96.59 ± 3.03
3	Cyanidin 3-*O*-galactoside	2.96	279,517	449^+^	287	2.64 ± 0.01
4	Cyanidin 3-*O*-arabinoside	3.12	279,512	419^+^	287	73.25 ± 2.23
5	Peonidin 3-*O*-glucoside	3.42	279,512	463^+^	301	118.46 ± 1.88
6	Peonidin 3-*O*-galactoside	3.56	278,519	463^+^	301	13.30 ± 0.22
7	Peonidin 3-*O*-arabinoside	3.71	279,517	433^+^	301	56.98 ± 0.61
8	Peonidin 3-*O*-xyloside	3.87	279,524	433^+^	301	2.85 ± 0.09
*Other phenolics*						
9	5-*O*-Caffeoylquinic acid	2.35	319	353^−^	191	4.82 ± 0.02
10	Caffeic acid *O*-glucoside I	2.53	329	341^−^	179	9.13 ± 0.21
11	Caffeic acid *O*-glucoside II	2.86	312	341^−^	179	5.54 ± 0.19
12	3-*O*-Caffeoylquinic acid	2.95	324	353^−^	191	29.45 ± 0.89
13	Coumaric acid *O*-glucoside I	3.11	310	325^−^	163	56.65 ± 2.62
14	Coumaric acid *O*-glucoside II	3.40	312	325^−^	163	6.61 ± 0.36
15	3-*O*-Feruloylquinic acid	3.50	329	355^−^	193	24.23 ± 0.17
16	4-*O*-Caffeoylquinic acid	3.57	329	353^−^	161	16.90 ± 0.14
17	Myricetin 3-*O*-glucoside	3.96	257,355	479^−^	317	120.19 ± 1.30
18	*p*-coumaric acid	4.19	307	163^−^	119	7.07 ± 0.13
19	Myricetin 3-*O*-pentoside	4.43	257,352	449^−^	317	42.01 ± 0.47
20	Quercetin 3-*O*-glucoside	4.60	255,355	463^−^	301	227.16 ± 2.99
21	Laricitrin 3-*O*-glucoside	4.71	271,354	493^−^	331	25.25 ± 0.04
22	Quercetin 3-*O*-pentoside I	4.91	255,355	433^−^	301	31.44 ± 1.81
23	Coumaroyl-dihydromonotropein	5.02	310	537^−^	163,119	38.51 ± 1.21
24	Quercetin 3-*O*-pentoside II	4.91	255,355	433^−^	301	111.37 ± 0.25
25	Quercetin 3-*O*-rhamnoside	5.31	255,352	447^−^	301	90.45 ± 2.75
26	Syringetin 3-*O*-glucoside	5.37	271,352	507^−^	345	20.59 ± 0.33
27	Kaempferol 3-*O*-glucoside	5.82	267,352	447^−^	285	5.28 ± 0.09
28	Kaempferol 3-*O*-galactoside	5.95	265,350	447^−^	285	6.55 ± 0.07
29	Syringetin 3-*O*-pentoside	6.07	271,350	477^−^	345	4.76 ± 0.15
30	Quercetin 3-*O*-(6”*p*-coumaroyl)-glucoside	6.37	255,326	609^−^	463,301	2.06 ± 0.03
31	Quercetin	6.75	255,350	301^−^	-	9.82 ± 0.03
32	Quercetin 3-*O*-(6”-*p*-benzoyl)-glucoside	7.31	255,355	567^−^	463,301	9.99 ± 0.36

**Table 3:** Profile of volatile compounds in the obtained cranberry extract.

No.	RT [min]	Peak Share in the Chromatogram [%]	Ordinary Substance Name	Systematic Substance Name	No CAS
1	12.42	45.7	-	ethyl benzoate	93-89-0
2	12.63	trace	-	benzoic acid	65-85-0
3	12.86	trace	*α*-terpinyl acetate	2-(4-methyl-3-cyclohexenyl)isopropyl acetate	80-26-2
4	12.95	trace	dihydrocarveol	5-isopropenyl-2-methylcyclohexanol	22567-21-1
5	15.40	3.16		butyl benzoate	136-60-7
6	15.70	trace	aromadendrene	7-methylene-3,3,11-trimethyltricyclo[6.3.0.02.4]undecane	489-39-4
7	16.13	14.66	*β*-caryophyllene	trans-(1R,9S)-8-methylene-4,11,11-trimethylbicyclo [7.2.0]undec-4-ene	87-44-5
8	16.39	3.46	ledene	(1S,2R,4R,11R)-3,3,7,11-tetramethyltricyclo[6.3.0.02.4]undec-7-ene	21747-46-6
9	16.57	trace	*α*-humulene	2,6,6,9-tetramethyl-1,4,8-cycloundecatriene	6753-98-6
10	16.82	trace	*α*-amorphene	naphthalene, 1,2,4a,5,6,8a-hexahydro-4,7-dimethyl-1-(1-methylethyl)-, (1S,4aR,8aS)-	23515-88-0
11	16.91	7.08	*(E)*-germacrene D	(1E,6E)-1-methyl-5-methylidene-8-propan-2-ylcyclodeca-1,6-diene	23986-74-5
12	17.10	3.45	*α*-muurolene	(1S,4aS,8aR)-4,7-dimethyl-1-propan-2-yl-1,2,4a,5,6,8a-hexahydronaphthalene	31983-22-9
13	17.29	4.07	*(R)*-gamma-cadinene	(1R,4aS,8aS)-7-methyl-4-methylidene-1-propan-2-yl-2,3,4a,5,6,8a-hexahydro-1H-naphthalene	39029-41-9
14	17.38	7.53	delta-cadinene	naphthalene, 1,2,3,5,6,8a-hexahydro-4,7-dimethyl-1-isopropyl-	483-76-1
15	18.14	6.62	-	n-eicosane	112-95-8
16	21.38	4.22	homomenthyl salicylate	benzoic acid, 2-hydroxy-, 3,3,5-trimethylcyclohexyl ester	118-56-9
TOTAL		99.95%			

## Data Availability

Data are contained within the article.
